# WHY DO WE NEED AN INTERNATIONAL CONVENTION ON THE RIGHTS OF OLDER PEOPLE?

**DOI:** 10.1093/geroni/igz038.1375

**Published:** 2019-11-08

**Authors:** Mika Marumoto

**Affiliations:** HelpAge International, London, United Kingdom

## Abstract

This presentation discusses the increasing need for an international convention on the rights of older people. Such a convention would contextualize global, regional and national demographic shifts and identify gaps in existing international human rights laws, so as to better protect older persons’ rights to health and well-being. Persons aged 60 or above are expected to more than double from 2015 to reach 2 billion in 2050, with their proportion of the world population rising from 12% to 21%. By 2050, 80% of older persons are expected to live in societies that are currently labeled developing countries. Existing international human rights instruments fall short regarding pensions and protection from poverty. The presentation demonstrates ongoing global efforts, specifically through the UN Open-ended Working Group on Ageing, to set global rights-based standards, and the roles played by civil society organizations that use network approaches in advocating for the rights of older people.

